# Racial/Ethnic Disparities in HIV Preexposure Prophylaxis Among Men Who Have Sex with Men — 23 Urban Areas, 2017

**DOI:** 10.15585/mmwr.mm6837a2

**Published:** 2019-09-20

**Authors:** Dafna Kanny, William L. Jeffries, Johanna Chapin-Bardales, Paul Denning, Susan Cha, Teresa Finlayson, Cyprian Wejnert, National HIV Behavioral Surveillance Study Group, National HIV Behavioral Surveillance Study Group, Meaghan Abrego, Alia Al-Tayyib, Bridget Anderson, Narquis Barak, Jeremy M. Beckford, Barbara Bolden, Kathleen A. Brady, Mary-Grace Brandt, Meredith Brantley, Sarah Braunstein, Celestine Buyu, Rosalinda Cano, Sidney Carrillo, Jie Deng, Karen Diepstra, Rose Doherty, Anna Flynn, Colin Flynn, David Forrest, Danielle German, Sara Glick, Henry Godette, Vivian Griffin, Emily Higgins, Theresa Ick, Tom Jaenicke, Antonio D. Jimenez, Salma Khuwaja, Monina Klevens, Irene Kuo, Zaida Lopez, Yingbo Ma, Stephanie Masiello Schuette, Melanie Mattson, Shanell L. McGoy, David Melton, Sandra Miranda De León, Willie Nixon, Chrysanthus Nnumolu, Conall O’Cleirigh, Jenevieve Opoku, E. Roberto Orellana, Paige Padgett, Jonathon Poe, H. Fisher Raymond, Toyah Reid, Alexis Rivera, William T. Robinson, Yadira Rolón-Colón, Randi Rosack, Sean Schafer, Ekow Kwa Sey, Jennifer Shinefeld, Emma Spencer, Ashley Tate, Jeff Todd, Margaret Vaaler, Afework Wogayehu, Pascale Wortley

**Affiliations:** 1 Division of HIV/AIDS Prevention, National Center for HIV/AIDS, Viral Hepatitis, STD, and TB Prevention, CDC.; Nassau and Suffolk counties, New York; Denver, Colorado; Nassau and Suffolk counties, New York; New Orleans, Louisiana; New Orleans, Louisiana; Newark, New Jersey; Philadelphia, Pennsylvania; Detroit, Michigan; Memphis, Tennessee; New York City, New York; Virginia Beach, Virginia; San Diego, California; New York City, New York; Dallas, Texas; Virginia Beach, Virginia; Boston, Massachusetts; San Diego, California; Baltimore, Maryland; Miami, Florida; Baltimore, Maryland; Seattle, Washington; Newark, New Jersey; Detroit, Michigan; Detroit, Michigan; San Francisco, California; Seattle, Washington; Chicago, Illinois; Houston, Texas; Boston, Massachusetts; Washington, DC; Houston, Texas; Los Angeles, California; Chicago, Illinois; Denver, Colorado; Memphis, Tennessee; Atlanta, Georgia; San Juan, Puerto Rico; Miami,; Florida; Philadelphia, Pennsylvania; Boston, Massachusetts; Washington, DC; Portland, Oregon; Houston, Texas; Dallas, Texas; San Francisco, California; Virginia Beach, Virginia; New York City, New York; New Orleans, Louisiana; San Juan, Puerto Rico; Memphis, Tennessee; Portland, Oregon; Los Angeles, California; Philadelphia, Pennsylvania; Miami, Florida; Nassau and Suffolk counties, New York; Atlanta, Georgia; Dallas, Texas; Newark, New Jersey; Atlanta, Georgia.

In 2017, preliminary data show that gay, bisexual, and other men who have sex with men (MSM) accounted for 67% of new diagnoses of human immunodeficiency virus (HIV) infection, that MSM who inject drugs accounted for an additional 3%, and that African American/black (black) and Hispanic/Latino (Hispanic) MSM were disproportionately affected (*1*). During 2010–2015, racial/ethnic disparities in HIV incidence increased among MSM; in 2015, rates among black and Hispanic MSM were 10.5 and 4.9 times as high, respectively, as the rate among white MSM (compared with 9.2 and 3.8 times as high, respectively, in 2010) (*2*). Increased use of preexposure prophylaxis (PrEP), which reduces the risk for sexual acquisition of HIV infection by approximately 99% when taken daily as prescribed,* would help to reduce these disparities and support the Ending the HIV Epidemic: A Plan for America initiative^†^ (*3*). Although PrEP use has increased among all MSM since 2014 (*4*), racial/ethnic disparities in PrEP use could increase existing disparities in HIV incidence among MSM (*5*). To understand racial/ethnic disparities in PrEP awareness, discussion with a health care provider, and use (steps in the HIV PrEP continuum of care) (*6*), CDC analyzed 2017 National HIV Behavioral Surveillance (NHBS) data. Black and Hispanic MSM were significantly less likely than were white MSM to be aware of PrEP, to have discussed PrEP with a health care provider, or to have used PrEP within the past year. Among those who had discussed PrEP with a health care provider within the past year, 68% of white MSM, 62% of Hispanic MSM, and 55% of black MSM, reported PrEP use. Prevention efforts need to increase PrEP use among all MSM and target eliminating racial/ethnic disparities in PrEP use.^§^

Data from CDC’s NHBS collected among MSM in 23 U.S. urban areas in 2017^¶^ (*7*) were analyzed to assess racial/ethnic disparities along the HIV PrEP continuum of care. The analysis was limited to participants at risk for HIV infection who likely met clinical indications for PrEP.** Men with a likely indication for PrEP included those who had 1) a negative NHBS HIV test result following the NHBS interview^††^; 2) either multiple male sex partners or any male sex partner with HIV infection within the past year; and 3) either condomless anal sex or a bacterial sexually transmitted infection^§§^ within the past year. Participants were asked whether they were aware of PrEP, had discussed PrEP with a health care provider, and had used PrEP within the past year.^¶¶^ Log-linked Poisson regression models with generalized estimating equations clustered on recruitment event and adjusted for urban area were used to estimate adjusted prevalence ratios (aPRs) and 95% confidence intervals (CIs). Analyses were conducted using SAS software (version 9.4; SAS Institute).

In 2017, a total of 10,104 sexually active MSM were interviewed in 23 U.S. urban areas. This analysis included 4,056 (40%) MSM (1,843 white MSM, 1,251 Hispanic MSM, and 962 black MSM) who tested negative for HIV and likely met the clinical indications for PrEP. Overall 1,742 (95%) white, 1,088 (87%) Hispanic, and 825 (86%) black MSM were aware of PrEP (white versus Hispanic aPR = 1.1, 95% CI = 1.0–1.1; white versus black aPR = 1.1, 95% CI = 1.0–1.1) (Figure). However, only 1,063 (58%) white, 546 (44%) Hispanic, and 412 (43%) black MSM reported discussing PrEP with a health care provider within the past year (white versus Hispanic aPR = 1.2, 95% CI = 1.1–1.3; white versus black aPR = 1.2, 95% CI = 1.1–1.3). Moreover, only 765 (42%) white, 373 (30%) Hispanic, and 248 (26%) black MSM reported taking PrEP within the past year (white versus Hispanic aPR = 1.2, 95% CI = 1.1–1.3; white versus black aPR = 1.4, 95% CI = 1.2–1.6). White MSM were significantly more likely than were Hispanic and black MSM to report PrEP awareness, discussion with a health care provider, and use.

**FIGURE:**
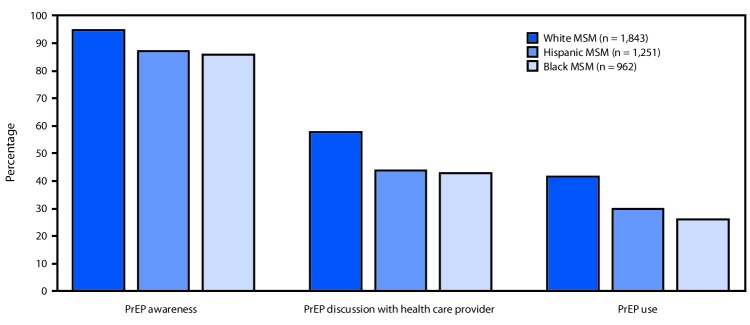
Preexposure prophylaxis (PrEP) awareness,* discussion,^†^ and use,^§^ by race/ethnicity, among men who have sex with men (MSM) with a likely indication for PrEP use^¶^ (N = 4,056) — 23 urban areas, 2017 **Abbreviations:** HIV = human immunodeficiency virus; NHBS = National HIV Behavioral Surveillance. * Respondents with a negative NHBS HIV test result were asked “Preexposure prophylaxis, or PrEP, is an antiretroviral medicine, such as Truvada, taken for months or years by a person who is HIV-negative to reduce the risk of getting HIV. Before today, have you ever heard of PrEP?” ^†^ If respondent had heard of PrEP before today, he was asked “In the past 12 months, have you had a discussion with a health care provider about taking PrEP?” ^§^ If respondent had heard of PrEP before today, he was asked “In the past 12 months, have you taken PrEP to reduce the risk of getting HIV?” ^¶^ Men with a likely indication for PrEP included those who had 1) a negative NHBS HIV test result following the NHBS interview; 2) either multiple male sex partners or any male sex partner with HIV infection within the past year; and 3) either condomless anal sex or a bacterial sexually transmitted infection within the past year.

Among 2,021 MSM who discussed PrEP with their health care provider, 225 of 412 (55%) black MSM used PrEP, compared with 338 of 546 (62%) Hispanic MSM and 724 of 1,063 (68%) white MSM (Table). White MSM who discussed PrEP with their health care provider were significantly more likely than were black MSM to use PrEP (aPR = 1.2, 95% CI = 1.1–1.3). This disparity between white and black MSM persisted among those who had health insurance (aPR = 1.2, 95% CI = 1.1–1.3) and had a usual source of health care (aPR = 1.2, 95% CI = 1.1–1.3), which are typical barriers to accessing prescription medication. Disparities in PrEP use between white and black MSM existed in the south (aPR = 1.2, 95% CI = 1.1–1.4) and west (aPR = 1.3, 95% CI = 1.0–1.6) U.S. census regions, whereas disparities between white and Hispanic MSM existed only in the south (aPR = 1.2, 95% CI = 1.1–1.4).

**TABLE:** Preexposure prophylaxis (PrEP) use among men who have sex with men (MSM) with a likely indication for PrEP use* who have discussed PrEP with a health care provider within the past 12 months (N = 2,021), by race/ethnicity and demographic characteristics — 23 urban areas, 2017

Characteristic	Black MSM		Hispanic MSM		White MSM		White MSM vs. black MSM		White MSM vs. Hispanic MSM	
	No. (%)	Total	No. (%)	Total	No. (%)	Total	aPR^†^	(95% CI)	aPR^†^	(95% CI)
**Overall**	**225 (54.6)**	**412**	**338 (61.9)**	**546**	**724 (68.1)**	**1,063**	**1.20**	**(1.08–1.32)**	**1.06**	**(0.98–1.14)**
**Age group (yrs)**										
18–24	57 (52.3)	109	55 (53.4)	103	71 (55.9)	127	1.03	(0.81–1.32)	1.03	(0.81–1.32)
25–34	116 (57.7)	201	190 (66.0)	288	334 (70.0)	477	1.17	(1.03–1.35)	1.04	(0.94–1.14)
35–44	38 (50.7)	75	67 (62.0)	108	189 (78.4)	241	1.48	(1.16–1.89)	1.21	(1.03–1.42)
≥45	14 (51.9)	27	26 (55.3)	47	130 (59.6)	218	1.09	(0.76–1.58)	0.96	(0.73–1.26)
**Education**										
Less than high school diploma	51 (47.2)	108	47 (57.3)	82	71 (67.0)	106	1.35	(1.06–1.73)	1.11	(0.89–1.40)
Some college or vocational school	79 (53.0)	149	127 (61.7)	206	156 (61.2)	255	1.13	(0.94–1.36)	0.98	(0.85–1.14)
College degree or graduate studies	94 (61.0)	154	164 (63.6)	258	497 (70.8)	702	1.13	(0.99–1.30)	1.07	(0.96–1.18)
**Household income**										
<$25,000	79 (53.0)	149	72 (57.1)	126	83 (58.9)	141	1.09	(0.87–1.36)	0.98	(0.79–1.20)
$25,000–$49,999	54 (46.2)	117	109 (64.1)	170	155 (66.0)	235	1.39	(1.11–1.73)	1.01	(0.87–1.16)
$50,000–$74,999	42 (62.7)	67	69 (60.5)	114	165 (67.9)	243	1.06	(0.86–1.31)	1.09	(0.92–1.30)
≥$75,000	50 (66.7)	75	87 (64.9)	134	321 (72.3)	444	1.07	(0.91–1.27)	1.07	(0.94–1.23)
**Currently have health insurance**										
No	27 (40.9)	66	37 (50.0)	74	43 (45.3)	95	1.11	(0.76–1.61)	0.89	(0.64–1.23)
Yes	198 (57.2)	346	301 (63.8)	472	681 (70.4)	968	1.19	(1.07–1.32)	1.07	(0.98–1.15)
**Usual source of health care when sick or need advice**										
No	23 (34.8)	66	33 (49.3)	67	30 (44.1)	68	1.22	(0.80–1.87)	0.84	(0.58–1.22)
Yes	202 (58.4)	346	303 (63.8)	475	692 (69.8)	991	1.17	(1.05–1.29)	1.06	(0.98–1.15)
**Bacterial STI within the past 12 mos**										
No	128 (51.8)	247	182 (54.7)	333	430 (63.6)	676	1.18	(1.03–1.34)	1.12	(1.00–1.25)
Yes	97 (58.8)	165	156 (73.2)	213	294 (76.2)	386	1.25	(1.09–1.44)	1.00	(0.91–1.10)
**Anal sex without a condom within the past 12 mos**										
No	10 (41.7)	24	7 (70.0)	10	12 (50.0)	24	1.18	(0.63–2.18)	0.68	(0.36–1.27)
Yes	215 (55.4)	388	330 (61.7)	535	712 (68.5)	1,039	1.18	(1.07–1.31)	1.07	(0.99–1.16)
**HIV status of last sex partner**										
Concordant	124 (51.7)	240	202 (61.0)	331	450 (68.0)	662	1.27	(1.11–1.45)	1.09	(0.99–1.20)
Discordant	32 (76.2)	42	36 (80.0)	45	65 (74.7)	87	0.96	(0.77–1.20)	0.92	(0.74–1.13)
Don't know HIV status	68 (53.1)	128	99 (59.3)	167	207 (66.6)	311	1.16	(0.96–1.40)	1.04	(0.90–1.20)
**Region** ^§^										
Midwest	21 (55.3)	38	21 (63.6)	33	63 (72.4)	87	1.11	(0.80–1.53)	1.14	(0.86–1.52)
Northeast	64 (55.2)	116	57 (66.3)	86	139 (65.3)	213	1.11	(0.90–1.37)	0.96	(0.80–1.17)
South	99 (54.7)	181	87 (54.0)	161	194 (67.8)	286	1.23	(1.06–1.42)	1.21	(1.02–1.43)
U.S. territories	0 (0.0)	0	4 (19.0)	21	1 (100.0)	1	N/A	N/A	N/A	N/A
West	41 (53.2)	77	169 (69.0)	245	327 (68.7)	476	1.28	(1.03–1.59)	1.00	(0.91– 1.10)

**Abbreviations:** aPR = adjusted prevalence ratio; CI = confidence interval; HIV = human immunodeficiency virus; N/A = not applicable; STI = sexually transmitted infection.

* Men with a likely indication for PrEP included those who had 1) a negative NHBS HIV test result following the NHBS interview; 2) either multiple male sex partners or any male sex partner with HIV infection within the past year; and 3) either condomless anal sex or a bacterial sexually transmitted infection within the past year.

^†^ aPRs were calculated using log-linked Poisson regression models with generalized estimating equations clustered on recruitment event and adjusted for urban area.

^§^ *Midwest region* includes Chicago, IL and Detroit, MI. *Northeast region* includes Boston, MA; Nassau and Suffolk counties, NY; New York City, NY; Newark, NJ; and Philadelphia, PA. *South region* includes Atlanta, GA; Baltimore, MD; Dallas, TX; Houston, TX; Miami, FL; New Orleans, LA; Virginia Beach, VA; and Washington, DC. *U.S. territories region* includes San Juan, PR. *West region* includes Denver, CO; Los Angeles, CA; Portland, OR; San Diego, CA; San Francisco, CA; and Seattle, WA.

## Discussion

In 2017, the disparities along the HIV PrEP continuum of care among black, Hispanic, and white MSM emerged at the point of discussion with a health care provider. Among those who discussed PrEP with their health care provider, disparities in PrEP use existed between black and white MSM. These findings highlight the need to address racial/ethnic disparities in PrEP awareness, discussions with health care providers, and, importantly, use among MSM. Black and Hispanic MSM currently experience substantially higher HIV incidence than do white MSM (*2*). Because PrEP effectively prevents sexual HIV transmission (*3*), racial/ethnic disparities in PrEP use might further increase disparities in HIV incidence (*5*). Social, structural, and epidemiologic factors are the underlying determinants of racial/ethnic health disparities (*8*). Therefore, prevention efforts that address these factors have the potential to decrease disparities along the HIV PrEP continuum of care.

Because disparities emerged at the point of discussion with a health care provider, interventions that increase PrEP awareness and discussion should include both patients and health care providers. As part of its Act Against AIDS campaign (now known as Let’s Stop HIV Together***), CDC disseminated Start Talking. Stop HIV,^†††^ which encourages MSM to discuss PrEP with their health care providers and sexual partners. Efforts that increase jurisdiction-level use of this campaign, especially in black and Hispanic communities, could help to increase awareness, discussion, and use of PrEP.

Although many social and structural factors that exacerbate racial/ethnic health disparities also create barriers to accessing health care, all persons with access to health care should have equal access to treatment. PrEP use among those without health insurance was relatively low across racial/ethnic subgroups. A recent agreement by Gilead Sciences, Inc. to donate PrEP medication to 200,000 uninsured persons at risk for HIV per year, is expected to help close the health care access gap.^§§§^ However, among MSM who discussed PrEP with their health care provider, the white versus black disparity in PrEP use persisted, even among MSM with health insurance. This finding suggests that black MSM face additional barriers to PrEP use beyond access to health care. Providers might make clinical decisions derived from inaccurate assumptions about racial/ethnic minority patients (*9*). This phenomenon can coalesce with patients’ mistrust of health care providers and inhibit discussions about PrEP and, ultimately, use of PrEP among black and Hispanic MSM. Health care provider trainings to promote PrEP discussions might address perceptions and assumptions that often limit their likelihood of discussing PrEP with MSM patients, especially black MSM (*9*). Health care providers could also benefit from culturally tailored trainings on taking a sexual history, which is essential for identifying black and Hispanic MSM with PrEP indications. Academic detailing and training to increase the number of health care providers prescribing PrEP and to enhance quality of care for PrEP patients, particularly in black and Hispanic neighborhoods, will also be important in reducing disparities (*10*). Finally, community- and provider-level interventions that destigmatize PrEP use, reduce medical mistrust, and educate about the prevention benefits of PrEP could be invaluable for increasing PrEP use among black and Hispanic MSM (*10*).

The findings in this report are subject to at least five limitations. First, NHBS uses a 12-month period for assessing risk behaviors, whereas the clinical guidelines use a 6-month period. This analysis used having multiple sex partners within the past year as a proxy for a nonmonogamous relationship, but these partnerships might not have overlapped in time. Thus, the analysis might include some men without indications for PrEP use. Their inclusion in the denominator might result in NHBS underestimation of the percentage of men for whom PrEP is indicated who use PrEP. Second, because data were not weighted to account for the complex sampling methods used to recruit MSM, estimates might be biased by over- or underestimating subpopulations. Third, NHBS is not nationally representative and might not be generalizable to all U.S. urban areas, nonurban areas, or all MSM. Fourth, data on self-reported behaviors might be subject to recall and social desirability bias. Social desirability bias might lead to overreporting PrEP awareness, discussion, and use. Finally, NHBS does not collect data on renal function, and persons with abnormal renal function are considered to have contraindication to PrEP use. Thus, it was not possible to adjust for differences in use based on medical contraindications.

Protecting persons at risk for HIV through effective, proven interventions, such as PrEP, is a pillar of the nation’s Ending the HIV Epidemic: A Plan for America initiative (*3*). PrEP is a highly effective and underused prevention tool for all MSM at high risk for HIV. Further efforts to improve outcomes along the HIV PrEP continuum of care for all MSM and to address racial/ethnic disparities, particularly in discussion with a health care provider and use, will be critical to reducing persistent racial/ethnic disparities in HIV incidence. These actions would help achieve the nation’s goal of preventing new HIV infections.

**Summary**

**What is already known about this topic?**

Preexposure prophylaxis (PrEP) reduces the risk for sexual human immunodeficiency virus transmission by approximately 99%. In 2017, approximately one third of gay, bisexual, and other men who have sex with men (MSM) reported using PrEP.

**What is added by this report?**

Although PrEP awareness was high for all racial/ethnic groups, a lower percentage of black and Hispanic MSM than white MSM had discussed PrEP with a health care provider or had used PrEP within the past year.

**What are the implications for public health practice?**

To expand PrEP use, interventions to increase PrEP awareness, encourage health care providers to discuss PrEP, destigmatize PrEP use, and promote racial/ethnic equity in PrEP access are needed.

## Footnotes

* https://www.cdc.gov/hiv/pdf/risk/prep/cdc-hiv-prep-guidelines-2017.pdf.

† https://www.hiv.gov/federal-response/ending-the-hiv-epidemic/overview?s_cid=ht_endinghivinternet0002.

§ https://www.cdc.gov/hiv/risk/prep/index.html.

¶ NHBS is cross-sectional biobehavioral surveillance system conducted in U.S. urban areas with high HIV prevalence. In 2017, MSM in 23 urban areas (Atlanta, Georgia; Baltimore, Maryland; Boston, Massachusetts; Chicago, Illinois; Dallas, Texas; Denver, Colorado; Detroit, Michigan; Houston, Texas; Los Angeles, California; Memphis, Tennessee; Miami, Florida; Nassau and Suffolk counties, New York; New Orleans, Louisiana; New York, New York; Newark, New Jersey; Philadelphia, Pennsylvania; Portland, Oregon; San Diego, California; San Francisco, California; San Juan, Puerto Rico; Seattle, Washington; Virginia Beach, Virginia; and Washington, DC) were recruited using venue-based sampling. Eligible participants (men who were born male and identified as male, reported having ever had oral or anal sex with another man, resided in the interview urban area, and were aged ≥18 years) completed standardized questionnaires in English or Spanish administered in person by trained interviewers. All participants were offered anonymous HIV testing and incentives for the interview and HIV test. The type of incentive (cash or gift card) and amount varied by urban area according to formative assessment and local policy. A typical incentive included $25 for completing the interview and $25 for providing a specimen for HIV testing.

** NHBS data do not correspond directly with the criteria for PrEP indication in the clinical guidelines. The guidelines recommend that men use PrEP if they are without acute or established HIV infection, have had sex with a nonmonogamous male partner who has not recently tested HIV-negative, and have had at least one of the following: any anal sex without a condom within the past 6 months or a bacterial sexually transmitted infection (i.e., syphilis, gonorrhea, or chlamydia) diagnosed or reported within the past 6 months. NHBS data flag persons who are likely indicated for PrEP use because of behavior from a longer period (12 months versus 6 months) and use multiple sex partners in a year as a proxy for a nonmonogamous partner.

†† HIV testing was performed for participants who consented. Blood specimens were collected for rapid testing in the field or laboratory-based testing. A nonreactive rapid test result was considered negative. A reactive rapid test was confirmed with either a second rapid test in the field or supplemental laboratory-based testing, typically western blot or indirect immunofluorescence assay.

§§ Syphilis, gonorrhea, or chlamydia.

¶¶ Participants were informed that PrEP is an antiretroviral medicine taken for months or years by a person who is HIV-negative to reduce the risk of getting HIV. Three steps of the HIV PrEP continuum of care were assessed by race/ethnicity, using the following questions: “Before today, have you ever heard of PrEP?” Respondents who answered “Yes” were asked “In the past 12 months, have you had a discussion with a health care provider about taking PrEP?” and “In the past 12 months, have you taken PrEP to reduce the risk of getting HIV?”

*** https://www.cdc.gov/stophivtogether/index.html?CDC_AA_refVal=https%3A%2F%2Fwww.cdc.gov%2Factagainstaids%2Fabout %2Findex.html.

††† https://www.cdc.gov/actagainstaids/campaigns/starttalking/index.html.

§§§ https://www.hhs.gov/about/news/2019/05/09/trump-administration-secures-historic-donation-of-billions-of-dollars-in-hiv-prevention-drugs.html.
